# The Complex Cell Wall Composition of Syncytia Induced by Plant Parasitic Cyst Nematodes Reflects Both Function and Host Plant

**DOI:** 10.3389/fpls.2017.01087

**Published:** 2017-06-21

**Authors:** Li Zhang, Catherine J. Lilley, Mustafa Imren, J. Paul Knox, Peter E. Urwin

**Affiliations:** ^1^Faculty of Biological Sciences, University of LeedsLeeds, United Kingdom; ^2^Faculty of Agriculture and Natural Sciences, Abant Izzet Baysal UniversityBolu, Turkey

**Keywords:** cyst nematode, syncytium, plant cell wall, methyl-esterified homogalacturonan, arabinan, xyloglucan, xylan

## Abstract

Plant–parasitic cyst nematodes induce the formation of specialized feeding structures, syncytia, within their host roots. These unique plant organs serve as the sole nutrient resource for development and reproduction throughout the biotrophic interaction. The multinucleate syncytium, which arises through local dissolution of cell walls and protoplast fusion of multiple adjacent cells, has dense cytoplasm containing numerous organelles, surrounded by thickened outer cell walls that must withstand high turgor pressure. However, little is known about how the constituents of the syncytial cell wall and their conformation support its role during nematode parasitism. We used a set of monoclonal antibodies, targeted to a range of plant cell wall components, to reveal the microstructures of syncytial cell walls induced by four of the most economically important cyst nematode species, *Globodera pallida*, *Heterodera glycines*, *Heterodera avenae* and *Heterodera filipjevi*, in their respective potato, soybean, and spring wheat host roots. *In situ* fluorescence analysis revealed highly similar cell wall composition of syncytia induced by *G. pallida* and *H. glycines*. Both consisted of abundant xyloglucan, methyl-esterified homogalacturonan and pectic arabinan. In contrast, the walls of syncytia induced in wheat roots by *H. avenae* and *H. filipjevi* contain little xyloglucan but are rich in feruloylated xylan and arabinan residues, with variable levels of mixed-linkage glucan. The overall chemical composition of syncytial cell walls reflected the general features of root cell walls of the different host plants. We relate specific components of syncytial cell walls, such as abundant arabinan, methyl-esterification status of pectic homogalacturonan and feruloylation of xylan, to their potential roles in forming a network to support both the strength and flexibility required for syncytium function.

## Introduction

**FIGURE 1:**
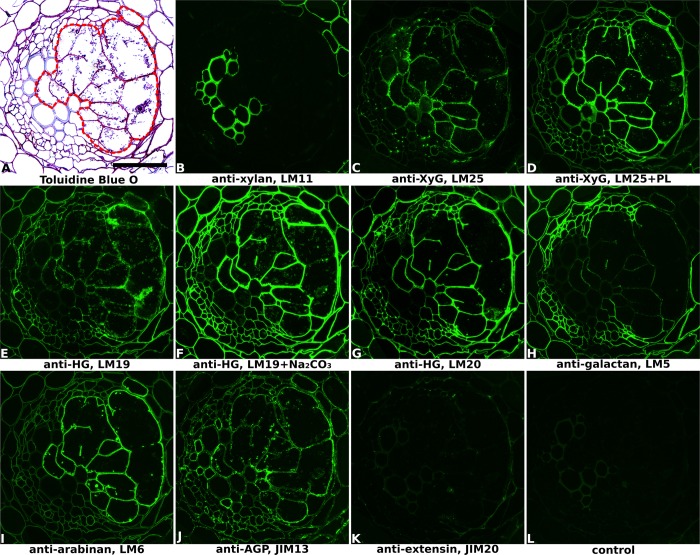
Immuno-fluorescence imaging of syncytia induced by potato cyst nematode *Globodera pallida* within potato roots (cv. Desiree, 14 dpi). **(A)** The extent of the syncytium is indicated in the Toluidine Blue O stained bright field image with a red line. Indirect immunofluorescence (green) resulting from the binding of specific mAbs is shown for corresponding serial sections: **(B)** LM11 to heteroxylan; **(C,D)** LM25 to xyloglucan (XyG); **(E,F)** LM19 to non/low methyl-esterified homogalacturonan (HG); **(G)** LM20 to methyl-esterified HG; **(H)** LM5 to pectic galactan; **(I)** LM6 to pectic arabinan; **(J)** JIM13 to AGPs; **(K)** JIM20 to extensin. LM11 binds only to the xylem vessels in the vascular cylinder **(B)** so serves to identify these cells in all sections. Control section **(L)** was processed without primary antibody. PL, pre-treated with pectate lyase; Na_2_CO_3_, pre-treated with Na_2_CO_3_; Scale bar = 50 μm.

**FIGURE 2:**
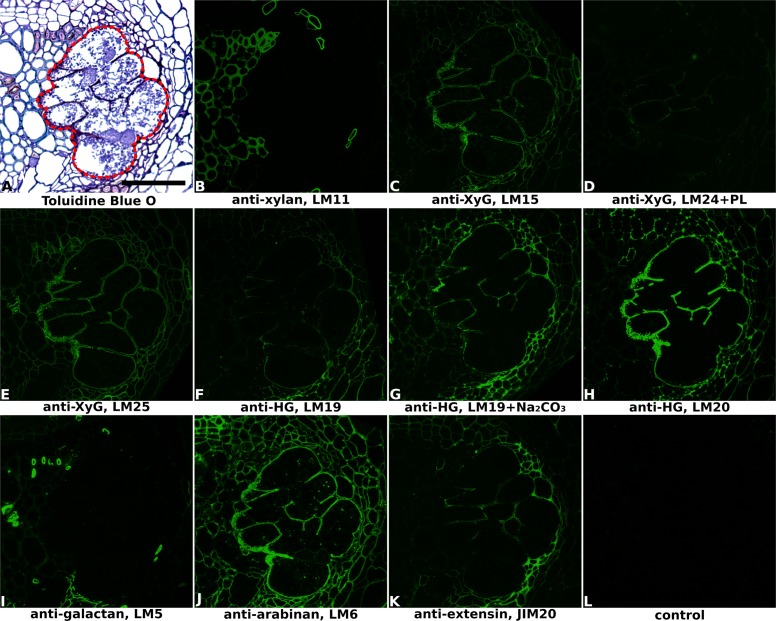
Immuno-fluorescence imaging of syncytia induced by soybean cyst nematode *Heterodera glycines* within soybean roots (cv. Toliman, 14 dpi). **(A)** The extent of the syncytium is indicated in the Toluidine Blue O stained bright field image with a red line. Indirect immunofluorescence (green) resulting from the binding of specific mAbs is shown for corresponding serial sections: **(B)** LM11 to heteroxylan; **(C)** LM15 to xyloglucan (XyG); **(D)** LM24 to xyloglucan (XyG); **(E)** LM25 to xyloglucan (XyG); **(F,G)** LM19 to non/low methyl-esterified homogalacturonan (HG); **(H)** LM20 to methyl-esterified HG; **(I)** LM5 to pectic galactan; **(J)** LM6 to arabinan; **(K)** JIM20 to extensin. LM11 binds only to the xylem vessels in the vascular cylinder **(B)** so serves to identify these cells in all sections. Control section **(L)** was processed without primary antibody. PL, pre-treated with pectate lyase; Na_2_CO_3_, pre-treated with Na_2_CO_3_; Scale bar = 50 μm.

Cyst nematodes are biotrophic sedentary endoparasites of plants that can establish prolonged parasitic interactions with their hosts, causing great economic losses worldwide (reviewed in Nicol et al., 2011). The most economically important cyst nematode species are from the *Globodera* and *Heterodera* genera, including potato cyst nematode (*Globodera rostochiensis* and *Globodera pallida*), soybean cyst nematode (*Heterodera glycines*) and cereal cyst nematode (CCNs) (*Heterodera avenae* and *Heterodera filipjevi*) (Jones et al., 2013). Cyst nematodes induce the formation of unique syncytial feeding structures as their sole nutrient resources, usually within the vascular cylinder of the host roots (see **Figures 1A**, **2A**, **4A,K**, **5A–D,M–P**). Infective second-stage juveniles (J2s) enter roots, generally in the zone of elongation, and migrate intracellularly toward the vascular cylinder. One initial syncytial cell (ISC), typically a single pericycle, procambial or inner cortical cell, is chosen and the induction of the syncytium is triggered after nematode pharyngeal gland cell secretions are injected into the ISC through the stylet. In the early stages of feeding site development neighboring cells fuse with the ISC through openings in the cell wall, formed by the widening of pre-existing plasmodesmata (Grundler et al., 1998). Once the syncytium has become established, plasmodesmata are no longer involved and there is local dissolution of the outer syncytium cell wall and that of neighboring cells (Ohtsu et al., 2017). The middle lamella is digested and, following fusion of the plasma membranes, the adjacent protoplasts are incorporated into the syncytium (Sobczak and Golinowski, 2009). Extensive hypertrophy of the syncytial elements, particularly closest to the nematode head, results in expansion of the feeding site. By consistently incorporating 100s of neighboring cells, the syncytium becomes a multinucleate nutrient sink and remains functional for the rest of the nematode life cycle (reviewed in Kyndt et al., 2013; Rodiuc et al., 2014). As the sole nutrient supply for nematode survival and development, the syncytium is metabolically highly active with prominent cytoplasm containing numerous plastids, mitochondria, proliferated endoplasmic reticulum, and other organelles (Bohlmann and Sobczak, 2014).

The process of syncytium initiation and formation is highly complex and the precise mechanisms are still largely unknown. It involves gene expression changes of both plant and nematode (Gheysen and Mitchum, 2009; Szakasits et al., 2009), cell wall degradation and restructuring (Bohlmann and Sobczak, 2014) and other related events (Goverse et al., 2000; Lilley et al., 2005; Davis et al., 2008). Fine microstructural/ultrastructural observations of forming syncytia have been accomplished for several different cyst nematode species in the last three decades, although little is yet known about the chemical composition of the cell walls (Sobczak and Golinowski, 2009). The syncytial cell wall microstructures will play a vital role in satisfying the functional requirements for successful nematode development. Plant cell walls are multifunctional polysaccharide-rich fibrous outer layers of plant cells, which can be divided into two types: primary walls of growing tissues and secondary walls of non-growing tissues (Fangel et al., 2012). The primary cell wall (PCW) is deposited during cell division and also during cell expansion and is composed of polysaccharides, proteins and aromatic substances. Typically, the PCW comprises cellulose microfibrils interlocked with cross-linking glycans, including mannans, xylans, xyloglucans and mixed-linkage glucans (MLG), depending on the plant species, further embedded in a pectin gel matrix. Pectic polysaccharide domains can be classified as homogalacturonan (HG), rhamnogalacturonan-I (RG-I), rhamnogalacturonan-II (RG-II), xylogalacturonan (XGA), and apiogalacturonan (AP) (Fangel et al., 2012; Atmodjo et al., 2013). Substantial differences exist between the cell wall composition and polymer structures of dicotyledons and commelinid monocotyledons, and even among the latter group there are large differences between the Poales (Poaceae, which include major crops) and others (Rose, 2003).

The composition of PCWs at cellular and sub-cellular levels can be determined using monoclonal antibodies that bind specifically to a range of cell wall polymers (Knox, 1997). This method has been used to investigate the cell wall components of different cell types in various plant species. Immunolabeling and fluorescence imaging of syncytia induced by *Heterodera schachtii* within *Arabidopsis thaliana* roots revealed that the distinct syncytial cell wall is composed of cellulose, xyloglucan, heteromannan, and methyl-esterified pectic homogalacturonan (methyl-HG) (Davies et al., 2012). However, this study focused on a model plant that is not an economic host for cyst nematodes and the chemical composition of syncytial cell walls induced by other cyst nematode species within their host roots is still unknown.

Here, we focus on the cell wall composition of syncytia induced by four of the most economically important cyst nematode species within their host roots: potato cyst nematode *G. pallida*, soybean cyst nematode *H. glycines* and CCNs *H. avenae* and *H. filipjevi*. Immuno-histochemical methods together with a set of monoclonal antibodies targeting various cell wall components were used to reveal the cell wall composition of syncytia formed in different host plants and determine features that are either conserved and therefore likely to be essential for syncytial function or are specific to a particular host–parasite interaction.

## Materials and Methods

### Plant Growth

Roots of potato (*Solanum tuberosum* cv. Desiree), soybean (*Glycine max* cv. Toliman) and three spring wheat cultivars (*Triticum aestivum* cv. Bobwhite, Cadenza, and Fielder) were infected with freshly hatched J2s of *G. pallida*, *H. glycines*, and CCNs (*H. avenae* and *H. filipjevi*) respectively.

Individual potato chits were removed from sprouting tubers and transferred to growth pouches (Mega International, St Louis Park, MN, United States). Soybean and wheat seedlings were similarly transplanted into pouches after germination on moist filter paper as described previously (Urwin et al., 2002). Cultivation was then carried out at 20°C (potato and wheat) or 25°C (soybean) under a 16 h/8 h photoperiod in a growth chamber (Sanyo MLR). Wheat seedlings were supplied with full-strength Hoagland nutrient solution (MP Biomedicals, Europe) once the third leaf was about to emerge. Water was added daily to replace evaporation losses and the nutrient solution was replaced after the first 2 weeks then subsequently once per week.

### Nematode Hatching and Inoculation

Second-stage juveniles of *G. pallida* and *H. glycines* were hatched from cysts in host root exudate at 20 or 25°C respectively and collected as described (Urwin et al., 1995). *H. avenae* and *H. filipjevi* cysts were rinsed in sterile tap water and stored in a 1.5 mL tube at 4°C for 1 month before hatching. Cysts were then placed into a sterile hatching jar and incubated at 10 or 4°C in the dark. Newly hatched J2s were collected every 2–3 days and could be stored at 4°C until required. At approximately 7 days after transfer to growth pouches, selected root tips of all plants were each inoculated with 20 J2s of the compatible nematode species and covered by a piece of GF/A paper (Sigma). Filter papers were removed 24 h post-inoculation. Infected and comparable non-infected regions of roots were excised at different time points post-infection.

### Probes for Cell Wall Analysis

Rat monoclonal antibodies used in this study were: LM10 and LM11 which bind to heteroxylan (McCartney et al., 2005), LM12 to feruloylated heteroxylan (Pedersen et al., 2012), LM28 to glucuronosyl-containing heteroxylan (Cornuault et al., 2015), LM15 to XXXG motif of xyloglucan (Marcus et al., 2008) and LM25 to XXXG/galactosylated xyloglucan (Pedersen et al., 2012), LM19 directed against low/non methyl-esterified HG (Verhertbruggen et al., 2009) and LM20 to highly methyl-esterified-HG (Verhertbruggen et al., 2009), LM5 to (1–4)-β-D-galactan (Jones et al., 1997), LM6 to (1–5)-α-L-arabinan (Willats et al., 1998), JIM20 to extensins (Smallwood et al., 1994; Knox et al., 1995) and MLG (Meikle et al., 1994).

### Immunolabeling and Fluorescence Imaging and Processing

Lengths of root harboring an established parasitic nematode and its associated syncytial feeding site were excised. Fixation and embedding together with subsequent sectioning and *in situ* analysis, were carried out using a described method (Davies et al., 2012). Serial transverse sections were collected from the mid-point of each syncytium to obtain the optimal size for downstream labeling and analysis. Any set of root sections that clearly harbored more than one syncytium was discarded. A minimum of five independent syncytia were sectioned and analyzed for each experiment with at least 24 technical replicate sections observed for each antibody. In order to eliminate background autofluorescence in wheat sections, a 5 min incubation using 0.1% Toluidine Blue O (pH 5.5, 0.2 M sodium phosphate buffer) was carried out after the immunolabeling steps. For bright field optical images, equivalent sections were stained with Toluidine Blue O solution (1% Toluidine Blue O dissolved in 1% sodium borate aqueous solution and filtered) for 5–10 min at room temperature then excess dye was washed off.

For unmasking of the LM25 xyloglucan epitope, pectic HG was enzymatically degraded with pectate lyase. Sections were first treated with 0.1 M sodium carbonate (pH 11.4) for 2 h at room temperature; washed with deionised water, then incubated in pectate lyase (*Aspergillus* sp.; Megazyme International, Ireland) at 25 μg/ml in 50 mM CAPS buffer with 1 mM CaCl_2_ (pH 10) for 2 h. Following three washes in deionised water immunolabeling was performed, as described in Davies et al. (2012).

Immunolabeled sections were observed under a Leitz DMRB Fluorescence Microscope [Leica Microsystems (UK) Ltd] and images were taken by QImaging QICAM digital camera (QImaging, Canada) using Q-capture pro software (QImaging, Canada). Further necessary image editing and composition was carried out using CorelDRAW X7 (Corel Corporation, Canada) and PaintShop Pro X7 (Corel Corporation, Canada). Fluorescence intensity measurements at 30–70 locations per cell type per antibody were carried out in ImageJ for the walls of the major host root cell types, including xylem, phloem elements, cortex and epidermis, as well as syncytial walls. For each antibody, the mean normalized value for each cell type was compared by one-way ANOVA and significant differences between syncytial walls and those of other cells were determined following Tukey’s multiple comparisons test.

## Results

### The Cell Walls of Syncytia Induced in Dicot Roots

Cell wall architectures of nematode-induced syncytia in dicotyledonous plants were analyzed using potato roots infected with *G. pallida* (**Figure 1** and Supplementary Figure S1) and soybean roots infected with *H. glycines* (**Figure 2** and Supplementary Figure S2), both at 14 days post-inoculation (14 dpi). Structural changes within the root associated with nematode parasitism were revealed by toluidine blue O staining of sectioned, fixed roots, and syncytial regions were outlined in bright-field images of each figure to aid interpretation. The xylan antibody LM11, which in dicots binds specifically to secondary cell walls, was used to visualize xylem vessels within vascular cylinders in both potato (**Figure 1B** and Supplementary Figure S1B) and soybean (**Figure 2B** and Supplementary Figure S2B) roots. This, together with the bright-field staining, allowed orientation of key root structures in spite of the gross changes in morphology that occurred during syncytium formation. The lower magnification images provided in Supplementary Figures S1, S2 allow the features of the syncytium to be viewed in context with the surrounding root cells.

The distribution of xyloglucan, the major non-cellulosic polysaccharide in dicots, was revealed mainly by the anti-xyloglucan probe LM25 in equivalent transverse sections through the syncytia of infected potato (**Figures 1C,D** and Supplementary Figures S1C,D) and soybean (**Figure 2E** and Supplementary Figure S2E) roots. The cell walls of syncytia induced by both *G. pallida* and *H. glycines* contained xyloglucans and the removal of pectic HG using pectate lyase was required to fully unmask the LM25 epitope in syncytial walls of potato (**Figure 1D**) similar to previous reports (Marcus et al., 2008; Davies et al., 2012). A further two probes which recognize different epitopes in xyloglucans (LM15 and LM24) also bound (data for soybean shown in **Figures 2C,D**, data for potato shown in Supplementary Figure S3) although the strongest binding was observed using LM25.

The presence of pectic HG, the major pectic polymer, was visualized using mAbs LM19 and LM20 (**Figures 1E–G**, **2F–H** and Supplementary Figures S1E–G, S2H,I), directed toward low/non methyl-esterified HG and highly methyl-esterified HG respectively. Differential binding of these two antibodies together with the effect of Na_2_CO_3_ pre-treatment to remove methyl-esters suggested that the syncytial cell wall of both potato and soybean possessed abundant pectic HG that was heavily methyl-esterified. LM20 bound strongly to syncytial cell walls in untreated sections (**Figures 1G**, **2H** and Supplementary Figures S1G, S2I) whereas LM19 only bound effectively following incubation with Na_2_CO_3_ (**Figures 1F**, **2G** and Supplementary Figure S1F). Strikingly different binding patterns of LM6 and LM5, recognizing 1,5-α-arabinan and 1,4-β-galactan side chains of pectic RG-I polysaccharides respectively, were observed in the two plant species. The LM6 arabinan epitopes were abundant within syncytial cell walls in both plants (**Figures 1I**, **2J** and Supplementary Figures S1I, S2G) while the LM5 galactan epitopes were barely detectable in walls of syncytia formed in potato (**Figure 1H** and Supplementary Figure S1H) and absent from those in soybean (**Figure 2I** and Supplementary Figure S2F).

In addition to polysaccharides, plant cell walls also possess various structural proteins. The JIM20 antibody-targeted epitope of extensin, a type of hydroxyproline-rich glycoprotein (HRGP), was detected at a low level in syncytial cell walls of soybean, especially the internal walls (**Figure 2K** and Supplementary Figure S2J) but was absent from walls of syncytia induced in potato (**Figure 1K** and Supplementary Figure S1K). Analysis of cell surface arabinogalactan-proteins (AGPs) using JIM13 showed that epitopes for this antibody were present within syncytial elements of potato (**Figure 1J** and Supplementary Figure S1J) but absent from those of soybean (data not shown).

At 14 dpi, the syncytia induced by female cyst nematodes have reached their maximum size (Urwin et al., 1997). The characteristics of cell walls during the extensive cell expansion and incorporation phase of syncytial formation may differ from those of a fully developed syncytium. Therefore we also analyzed sections of potato roots infested with juvenile *G. pallida* at 7 dpi when the syncytia were clearly less developed and still undergoing expansion (Supplementary Figure S4). The binding patterns of the monoclonal antibodies (Supplementary Figure S4) were very similar to those observed at 14 dpi (**Figure 1** and Supplementary Figure S1). This suggests that the syncytial cell walls maintain a consistent structural composition during the course of syncytium formation and nematode development.

**FIGURE 3:**
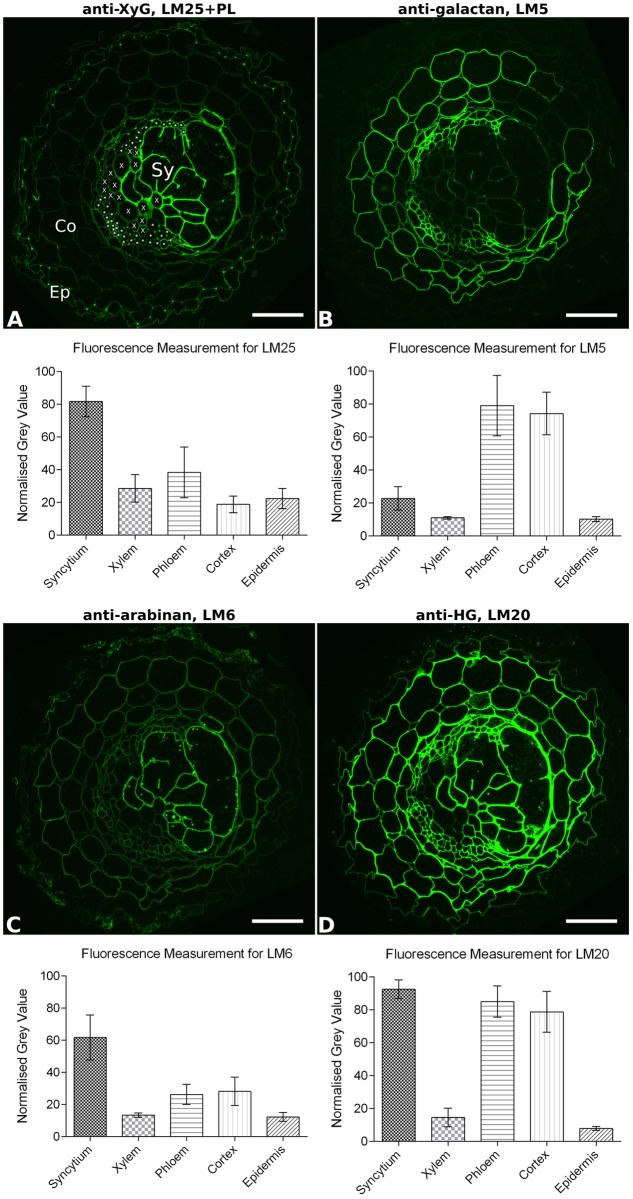
Quantitative fluorescence analysis of general cell wall polymers in transverse sections of potato root infested with *Globodera pallida* (14 dpi). Fluorescence (green) images show the specific binding of mAbs **(A)** LM25 to xyloglucan (XyG), **(B)** LM5 to galactan, **(C)** LM6 to arabinan, and **(D)** LM20 to methyl-esterified homogalacturonan (HG), on equivalent serial sections. To aid interpretation, the cell types analyzed are indicated in **(A)**: Ep, example epidermal cell; Co, example cortical cell; X, each xylem vessel, ^∗^ indicates each phloem element, Sy, syncytium. The extent of the syncytium can be most clearly visualized by the strong fluorescence in **(A,C)**. Mean normalized fluorescence measurements for each cell type (*n* = 30–70) are shown. Error bars represent standard deviation. Differences in fluorescence between cell walls of syncytia and all other cell types are significant for each antibody (*P* ≤ 0.001; one-way ANOVA with Tukey’s multiple comparisons test). PL, section pre-treated with pectate lyase; Scale bar = 50 μm.

The overall binding patterns of the various mAbs to syncytial cell walls within the context of the entire root section were also analyzed and shown in Supplementary Figure S1 for potato whole root and Supplementary Figure S2 for the soybean vascular cylinder. In comparison to the other root cells of the vascular cylinder, the cyst nematode-induced syncytia were seen to have a distinctive cell wall chemical composition. For example, syncytial cell walls lack xylan compared to host xylem elements (Supplementary Figures S1B, S2B), and contain more abundant xyloglucan (Supplementary Figures S1C,D, S2C–E) but lack galactan (Supplementary Figures S1H, S2F) compared to the phloem elements. The differential abundance of xyloglucan, galactan, arabinan, and methyl-esterified HG was investigated in more detail for the walls of syncytia, xylem, phloem, cortical, and epidermal cells in potato roots (**Figure 3**). A fluorescence quantification method was applied over the *G. pallida*-infested potato root sections, and the results show the clearly distinct nature of syncytial walls from walls of other major root cell types (**Figure 3**). Cell walls of syncytia contained significantly more xyloglucan detected by LM25 (**Figure 3A**) and arabinan detected by LM6 (**Figure 3C**) than those of any other cell type analyzed. The galactan content of the syncytium cell walls was significantly lower than for cortex or phloem (**Figure 3B**) but significantly higher than for either xylem vessels or epidermal cells. Of the four components analyzed, the widely present methyl-esterified HG was most similar between walls of the syncytium and other cell types (**Figure 3D**), however it was still significantly more abundant in the syncytium than either phloem or cortical cells. No other cell type in the potato roots displayed an identical wall composition to syncytia, confirming the unique nature of the syncytium.

### The Cell Walls of Syncytia Induced by *H. avenae* and *H. filipjevi* in Wheat Roots

The globally important wheat crop can be severely affected by CCNs (Nicol et al., 2011). We carried out a comprehensive analysis of cell wall structure encompassing syncytia induced by the two most economically important species *H. avenae* and *H. filipjevi* in three spring wheat cultivars (Bobwhite, Cadenza, and Fielder) at different stages of the infection process. Roots were infected with J2s of *H. avenae* and *H. filipjevi*, and samples were collected when similar stages of adult females of both nematode species were observed on the root surface (21 dpi for *H. avenae* and 28 dpi for *H. filipjevi*).

**FIGURE 4:**
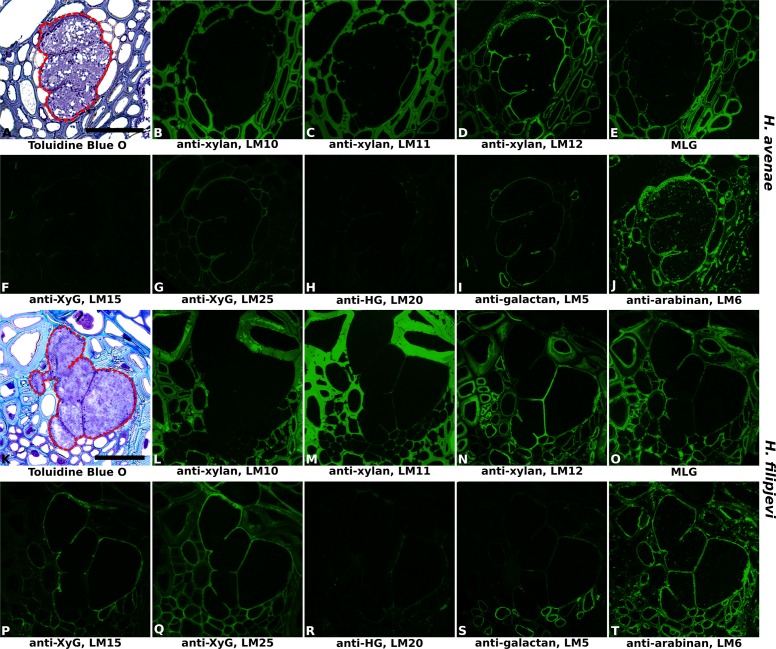
Immuno-fluorescence imaging of syncytia induced by the cereal cyst nematodes (CCNs) *Heterodera avenae* and *Heterodera filipjevi* within wheat roots (cv. Cadenza, 21 dpi and 28 dpi respectively). **(A,K)** Bright field images of Toluidine Blue O stained sections. The extent of the syncytium is indicated with a red line. Indirect immunofluorescence (green) resulting from the binding of specific mAbs is shown for corresponding serial sections: **(B,L)** LM10 to heteroxylan; **(C,M)** LM11 to heteroxylan; **(D,N)** LM12 to feruloylated heteroxylan; **(E,O)** MLG to mixed linkage glucan (MLG); **(F,P)** LM15 to xyloglucan (XyG); **(G,Q)** LM25 to xyloglucan (XyG); **(H,R)** LM20 to methyl-esterified homogalacturonan (HG); **(I,S)** LM5 to pectic galactan; **(J,T)** LM6 to arabinan. Scale bar = 50 μm.

Heteroxylans are the major cross-linking glycans in PCWs of grasses. Three monoclonal antibodies (LM10, LM11 and LM12) that recognize unsubstituted xylan, unsubstituted xylan/arabinoxylan and feruloylated xylan respectively were used to reveal both the presence and the substitutions of xylans in syncytial cell walls. Antibody binding patterns revealed the absence of heteroxylan recognized by LM10 (**Figures 4B,L** and Supplementary Figures S5B,L, S6B,L) and LM11 (**Figures 4C,M** and Supplementary Figures S5C,M, S6C,M) within syncytial cell walls. Strong binding was observed for LM12 (**Figures 4D,N** and Supplementary Figures S5D,N, S6D,N), suggesting that syncytial cell wall xylans are extensively substituted with ferulic acid. The xyloglucan epitopes recognized by LM15 and LM25 were present within syncytial walls induced by both nematode species, with that of LM25 relatively more abundant (**Figures 4F,G,P,Q** and Supplementary Figures S5F,G,P,Q, S6F,G,P,Q). MLG is mainly restricted to the grass family and was evident in a range of cell types in the root sections. However, whilst it was present in the cell walls of syncytia induced by *H. filipjevi* (**Figure 4O** and Supplementary Figures S6E,O), it was almost entirely absent from *H. avenae*-induced syncytia (**Figure 4E** and Supplementary Figures S5E,O).

The prevalence of pectic polysaccharides in the syncytial cell walls of wheat was analyzed using LM19, LM20, LM5, and LM6. In contrast to the syncytia formed in potato and soybean, only trace levels of methyl-esterified HG were observed, indicated by the slight binding of LM20 (**Figures 4H,R** and Supplementary Figures S5H,R, S6H,R). However, this was clearly due to a lack of HG, rather than an altered methyl-esterification status, as the LM19 epitope was entirely absent (data not shown). Low levels of galactan were detected with LM5 (**Figures 4I,S** and Supplementary Figures S5I,S, S6I,S) whilst arabinan epitopes detected by LM6 were much more abundant (**Figures 4J,T** and Supplementary Figures S5J,T, S6J,T).

**FIGURE 5:**
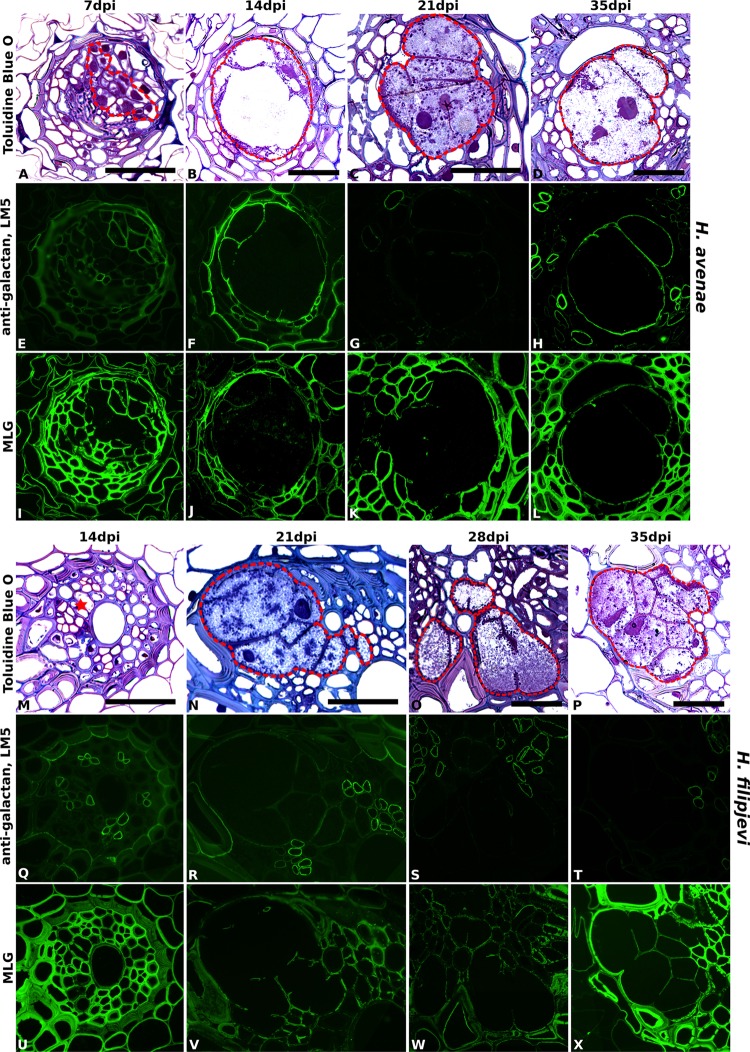
Immuno-fluorescence imaging of galactan and MLG in syncytia induced by *H. avenae* and *H. filipjevi* within wheat roots (cv. Bobwhite). (**A–D** for *H. avenae* and **M–P** for *H. filipjevi*) Bright field images of Toluidine Blue O stained sections. Syncytial regions are outlined in red except for **(M)** where the likely developing syncytial region is denoted by a red asterisk. Indirect immunofluorescence (green) resulting from the binding of (**E–H** for *H. avenae* and **Q–T** for *H. filipjevi*) mAb LM5 to pectic galactan and (**I–L** for *H. avenae* and **U–X** for *H. filipjevi*) mAb MLG to mixed linkage glucan is shown for different time points after infection as indicated. Scale bar = 50 μm.

Importantly, no differences in antibody binding patterns (for instance, abundant vs. absent) were observed between cell walls of syncytia induced by the same nematode species in the three wheat cultivars. Therefore, for investigation of temporal changes in cell wall composition during nematode and syncytium development, sections of syncytia induced by *H. avenae* and *H. filipjevi* in only the cultivar Bobwhite were analyzed. The antibody binding was generally stable in syncytia induced by the same nematode species among different stages (Supplementary Figure S7 for *H. avenae* and Supplementary Figure S8 for *H. filipjevi*) and syncytia induced by both nematode species shared high similarities in cell wall chemical composition, apart from LM5 and MLG (**Figure 5**). The feruloylated heteroxylan in syncytial walls also contained glucuronic acid decorations (indicated by LM28, data for *H. avenae* shown in Supplementary Figure S7), indicating the abundance of xylan substitutions in walls of syncytia induced by both nematode species. The LM5 galactan epitope was largely absent from *H. filipjevi*-induced syncytial walls among all the stages analyzed (**Figures 5Q–T**) whilst shown to be present in *H. avenae*-induced syncytia at certain stages (**Figures 5E–H**). The previously observed absence of MLG in syncytia of *H. avenae* and presence in those of *H. filipjevi* MLG was most likely not related to differences in syncytial development. MLG was barely detectable in syncytia of *H. avenae* at a range of developmental stages (**Figures 5I–L**) whilst it remained present in all syncytial stages of *H. filipjevi* (**Figures 5U–X**).

The cell walls of syncytia induced by both *H. avenae* and *H. filipjevi* in wheat roots were rich in highly substituted heteroxylan and arabinan and also contained xyloglucan. Syncytia induced by the same nematode species had generally conserved cell wall chemical compositions, both among different host cultivars and among various nematode developmental stages within the same cultivar. Apart from the different binding of pectic galactan and MLG, syncytia of both nematode species in wheat possess similar cell wall microstructures. As analyzed using the same host at a series of developmental stages, those differences of galactan and MLG are more likely related to the nematode species although the precise reasons are yet to be elucidated.

## Discussion

Cellulose microfibrils provide the scaffolding and rigidity of cell walls and they are cross-linked with hemicelluloses, including mannans, xylans, MLG, and xyloglucans. This network is further embedded in a matrix of pectic polysaccharides (Scheller and Ulvskov, 2010; Fangel et al., 2012). These complex non-cellulosic glycans can form various linkages, thus affecting the mechanical properties of cell walls and reflecting the growth status of plants. Therefore, revealing the chemical composition of syncytial walls can provide insight into how this unique feeding structure fulfills its role during cyst nematode parasitism, as the syncytial cell walls must be capable of generating sufficient mechanical strength to withstand the much higher turgor pressure within (Böckenhoff and Grundler, 1994) whilst maintaining the necessary flexibility to cope with the periodic demands of nematode feeding (Muller et al., 1981).

### Xyloglucan Is the Major Non-cellulosic Polymer in Both *G. pallida* and *H. glycines* Induced Syncytial Cell Walls, While Highly Substituted Heteroxylan Is Abundant in Walls of Syncytia Induced in Wheat

Xyloglucan has been found in almost every land plant species analyzed and is the most abundant hemicellulose in primary walls of spermatophytes except for grasses (Scheller and Ulvskov, 2010; Pauly et al., 2013). It was detected in the cell walls of all syncytia analyzed, irrespective of host or nematode species although, consistent with its distribution in the plant kingdom, it was more abundant in syncytia within potato and soybean roots than those formed in wheat roots. The function of xyloglucan in the plant cell wall is described by the load-bearing xyloglucan/cellulose framework model: increasing xyloglucan tethers between the cellulose microfibrils causes increased rigidity of the cell wall, while degradation of these tethers causes the walls to loosen (Pauly et al., 2013). The dynamic changes of xyloglucan cross-linking, catalyzed by enzymes such as xyloglucan endo-*trans*glycosylase/hydrolase (XTH), could mediate the cell wall restructuring that is required during syncytium formation. For instance, the expression of *Arabidopsis XTH9* and an endo-xyloglucan transferase *XTR6* were both up-regulated in syncytia of *H. schachtii*, whilst *XTR9* was down-regulated (Szakasits et al., 2009), reflecting the reconstruction of host cell walls during syncytium formation and functioning. Walls of syncytia in soybean also possess abundant xyloglucan, however, in this case a homolog of *Arabidopsis XTR6* was found to be slightly downregulated during the interaction with *H. glycines* (Ithal et al., 2007a). Several xyloglucan endo-*trans*glycosylase (*XET*) genes were highly upregulated at the early stage of syncytium induction by *H. glycines*, with transcripts of one gene localized in 5-day old syncytia (Ithal et al., 2007b). XET activity can also be involved in deposition of new wall material (Nishitani and Tominaga, 1992), therefore the dynamic change of this network, breaking down or generating new cross-links, may be regulated as the nematode develops.

Xylans, a diverse group of glycans, are the dominant non-cellulosic polysaccharide in the secondary cell walls of dicotyledonous plants. They are commonly substituted with glucuronosyl residues to form glucuronoxylans and previous research has shown that syncytia formed in *Arabidopsis* roots possess no secondary cell walls and correspondingly contain no xylans (Davies et al., 2012; Wieczorek et al., 2014). Similarly, no xylan epitopes were detected within syncytial cell walls in potato and soybean roots. Conversely, xylans are abundant in primary walls of commelinid monocots such as Poales including wheat, rice and maize, and usually contain many arabinose residues attached to the backbone, forming arabinoxylans (AXs) and glucuronoarabinoxylans (GAXs) (Buanafina, 2009; Scheller and Ulvskov, 2010). The AXs serve an important role by cross-linking cellulose microfibrils as well as oxidatively linking with each other. The walls of growing cells predominantly contain highly substituted AXs rather than the less branched xylans (Buanafina, 2009). Syncytial cell walls in wheat contained no un-/low-substituted xylans but a small amount of AX/GAX. The strong binding of LM28, targeting glucuronosyl-containing heteroxylans (Cornuault et al., 2015) also suggested high substitution of heteroxylans in walls of syncytia formed in wheat. Meanwhile LM12, derived to recognize feruloyl residues attached to a range of sugars (Pedersen et al., 2012), bound strongly within wheat syncytial cell walls, most likely to feruloylated xylans. Together, this indicates the importance of xylan substitution in both formation and function of syncytia in wheat. Ferulate esters can be oxidatively cross-linked in a variety of ways, potentially causing cell wall stiffening and reduced growth and expansion. Feruloylation is also correlated with cell wall degradability (Buanafina, 2009). Cell wall integrity was compromised in transgenic plants expressing a fungal feruloyl esterase *AnFAE* that caused a reduction of ferulic acids and the plants were more susceptible to fungal pathogens (Reem et al., 2016). A similar study has also shown the link between the expression of fungal ferulic acid esterase and cell wall digestibility (Badhan et al., 2014). The abundance of xylan substitutions in syncytial cell walls of wheat may indicate that various cross-links are formed to maintain syncytial wall integrity and provide mechanical strength. The reported near absence of heteroxylans in the walls of syncytia formed by *H. avenae* in barley, a host also with heteroxylan-rich primary walls (Aditya et al., 2015) may result from the use of only anti-xylan LM11 in that study. Further analysis with a wider range of probes that recognize substituted forms of xylan, would likely be informative.

### Pectin HG Is Highly Methyl-Esterified in Both Potato and Soybean Syncytial Cell Walls

Pectic polysaccharides, of which HG is the major group, are abundant in plant cell walls, comprising as much as 30% of dicot, gymnosperm, and non-Poales monocot cell walls, but considerably less in cell walls of grasses (Caffall and Mohnen, 2009). Homogalacturonan was abundant and predominantly methyl-esterified in the cell walls of syncytia induced by *G. pallida* and *H. glycines*. Interestingly, methyl-HG was detectable at a low level within several syncytial wall samples of wheat, despite that host generally lacking abundant pectin in PCWs. Thus, the methyl-esterification status of HG in cell walls of syncytia formed in potato, soybean and even in wheat is similar to that previously described for mature syncytia in *Arabidopsis* (Davies et al., 2012; Wieczorek et al., 2014) and barley (Aditya et al., 2015), although HG was reported to be largely unmethylesterified at very early stages of syncytium formation (Wieczorek et al., 2014).

Pectin HGs are highly methyl-esterified when initially deposited into cell walls and can subsequently be de-esterified by the action of pectin methylesterases (PMEs) to facilitate the formation of HG-calcium complexes, the so called ‘egg-box’ model (Caffall and Mohnen, 2009; Wolf et al., 2009). The presence of the HG-calcium structure is postulated to induce pectic gel formation and thus cause cell wall stiffening, therefore the abundant methyl-HG might contribute to wall flexibility required during nematode feeding (Muller et al., 1981; Bohlmann and Sobczak, 2014). The rheological properties of the syncytium cell wall, including its porosity and extensibility, could be modulated by spatially distributed modifying enzymes such as PMEs and PMEIs (PME inhibitors) (Wolf et al., 2009). A secreted cellulose-binding protein (HsCBP) from the nematode *H. schachtii* specifically interacts with *Arabidopsis* PME3 to facilitate cyst nematode parasitism. Its expression peaks at the parasitic J3 stage, suggesting a role during the early phase of syncytium formation (Hewezi et al., 2008). However, both PMEs and PMEIs are large gene families with 67 PMEs (PF01095, EC 3.1.1.11) in *Arabidopsis* (Lombard et al., 2014) and 69 PMEIs (Saez-Aguayo et al., 2013). Their complex activities and interactions in the syncytia are likely to be responsible for regulating the abundant pectin methyl-HG in the syncytial walls, thus facilitating nematode parasitism. Further investigations should be made to elucidate the methyl-HG accumulation mechanism in syncytial walls as well as its impact on syncytium mechanical properties, especially considering the innate complexity of the role of possible pectin cross-links (Peaucelle et al., 2012).

### Pectic Arabinans Are Abundant in All Analyzed Syncytial Cell Walls

The high abundance of RG-I pectic arabinan side chains was a striking common feature of all syncytial walls assessed, regardless of plant host or nematode species. This implies a conserved and important role, most likely to be in maintaining cell wall flexibility as reported for other specialized plant cell types. High arabinan content helps to maintain flexibility in guard cell walls (Jones et al., 2003), and is also implicated in the response of other plant cells facing water loss and therefore a change in cell volume (Moore et al., 2006, 2008). Due to the large volumes withdrawn by the feeding nematodes (Muller et al., 1981) and the high turgor pressure recorded inside syncytia (Böckenhoff and Grundler, 1994), in addition to the fluctuation in size during nematode development, such wall flexibility seems to be essential for general syncytial function.

In contrast to the abundant arabinan side chains, pectic galactan (indicated by LM5) was found to be absent from soybean root syncytia, as also shown previously for *Arabidopsis* (Davies et al., 2012), and was present at a very low level in walls of syncytia formed in potato and wheat. The higher natural abundance of galactan in potato (Oxenbøll Sørensen et al., 2000) may account for the difference among the dicot hosts. Pectic galactan may also be related to mechanical rigidity of cell walls (McCartney et al., 2000) although in this case, presence of pectic (1→4)-β-D-galactan correlates with firmer plant tissues. Therefore the relative absence of galactan residues is also likely to contribute to syncytial cell wall flexibility.

As a nutrient sink and the sole supply of food to the cyst nematode, the syncytium has a generally stable cell wall composition of complex polysaccharides during the nematode life cycle to maintain its function throughout parasitism. The substitutions and modifications of those cell wall polymers indicate that the syncytial wall is a dynamic, flexible structure, capable of fulfilling various requirements in plant–nematode interactions. Cell walls of syncytia are clearly distinct from those of surrounding root cells. Nevertheless, as a fusion of remodeled host plant cells, syncytial cell walls also possess key characteristics of their host plant cell walls: syncytia in potato, soybean and previously *Arabidopsis* hosts with dicot-type primary walls, contain abundant pectins and xyloglucans while syncytial cell walls in the commelinid monocot host wheat, possess large amounts of substituted heteroxylans, small amount of xyloglucans and very few pectic polysaccharides. This reflects the host-specific adaptations in syncytial formation, involving the use of existing cell wall synthesis mechanisms in host roots.

## Author Contributions

LZ, PU, JK conceived and designed the study; LZ carried out all experiments and analyzed data; MI provided materials and methodology; LZ, CL, JK, PU wrote the manuscript; All authors critically revised and approved the manuscript.

## Conflict of Interest Statement

The authors declare that the research was conducted in the absence of any commercial or financial relationships that could be construed as a potential conflict of interest.
