# Supplementary material for: The Complex Cell Wall Composition of Syncytia Induced by Plant Parasitic Cyst Nematodes Reflects Both Function and Host Plant
Source: Front Plant Sci. 2017 Jun 21;8:1087. doi: 10.3389/fpls.2017.01087 (PMC5478703; doi:10.3389/fpls.2017.01087)
Supplement: Supplementary file 1 [file Data_Sheet_1.PDF]

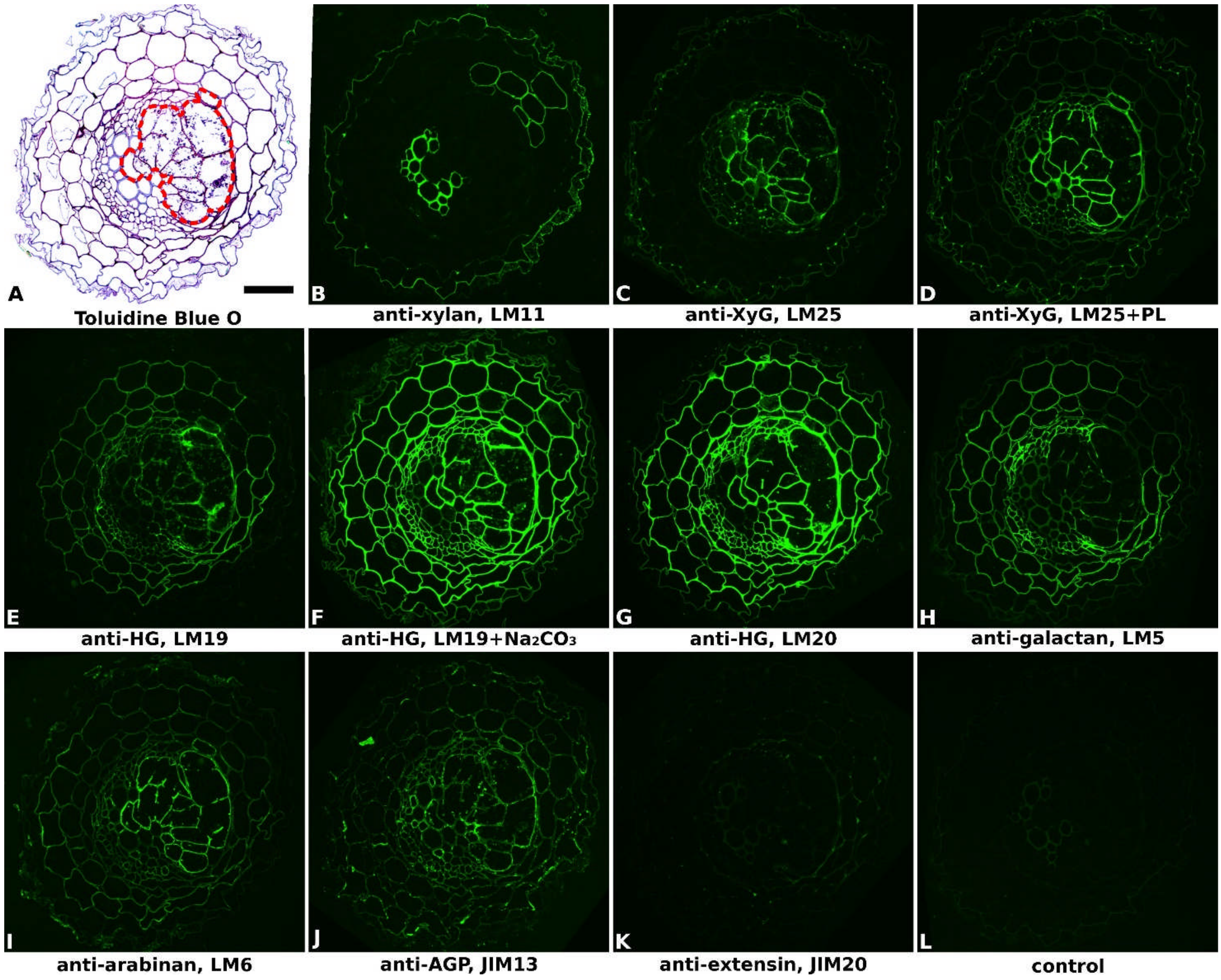

Figure S1. Immunofluorescence imaging of a sectioned potato root infected with *G. pallida* (cv Desiree, 14 dpi). (A) The extent of the syncytium is indicated in the Toluidine Blue O stained bright field image with a red line. Indirect immunofluorescence (green) resulting from the binding of specific mAbs is shown for corresponding serial sections: **(B)** LM11 to heteroxylan; **(C & D)** LM25 to xyloglucan (XyG); **(E & F)** LM19 to non/low methyl-esterified homogalacturonan (HG); **(G)** LM20 to methyl-esterified HG; **(H)** LM5 to pectic galactan; **(I)** LM6 to pectic arabinan; **(J)** JIM13 to AGPs; **(K)** JIM20 to extensin. Within the vascular cylinder LM11 binds only to the xylem vessels **(B)** so serves to identify the location of these cells in all sections. Control section **(L)** was processed without primary antibody. PL: pre-treated with pectate lyase; Na_2CO_3: pre-treated with Na_2CO_3; Scale bar = 50 µm.

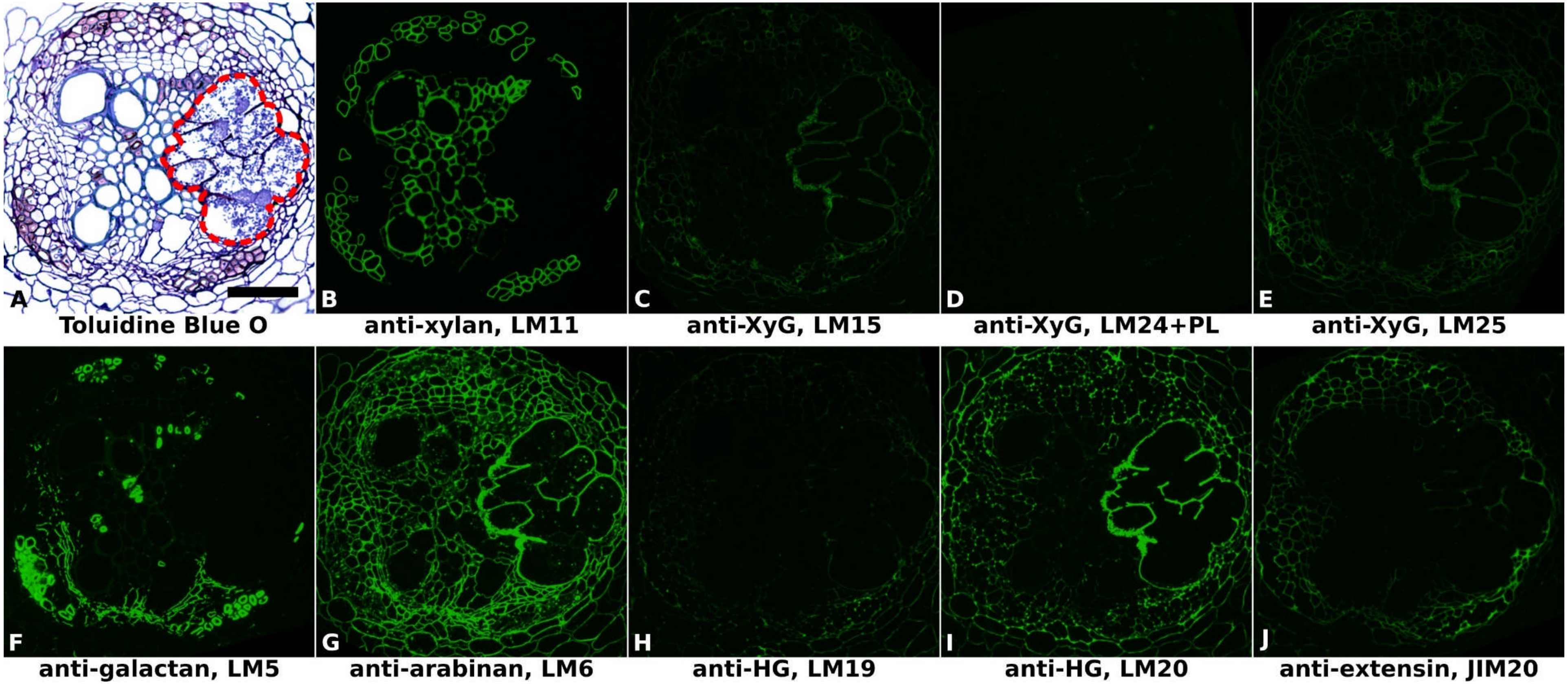

Figure S2. Immunofluorescence imaging of the vascular cylinder of a sectioned soybean root infected with *H. glycines* (cv Toliman, 14 dpi). (A) The extent of the syncytium is indicated with a red line in the Toluidine Blue O stained bright field image. Indirect immunofluorescence (green) resulting from the binding of specific mAbs is shown for corresponding serial sections: **(B)** LM11 to heteroxylan; **(C)** LM15 to xyloglucan (XyG); **(D)** LM24 to xyloglucan (XyG); **(E)** LM25 to xyloglucan (XyG); **(F)** LM5 to pectic galactan; **(G)** LM6 to arabinan; **(H)** LM19 to non/low methyl-esterified homogalacturonan (HG); **(I)** LM20 to methyl-esterified HG; **(J)** JIM20 to extensin. LM11 binds only to the xylem vessels in the vascular cylinder **(B)** so serves to identify these cells in all sections. PL: pre-treated with pectate lyase. Scale bar = 50 µm.

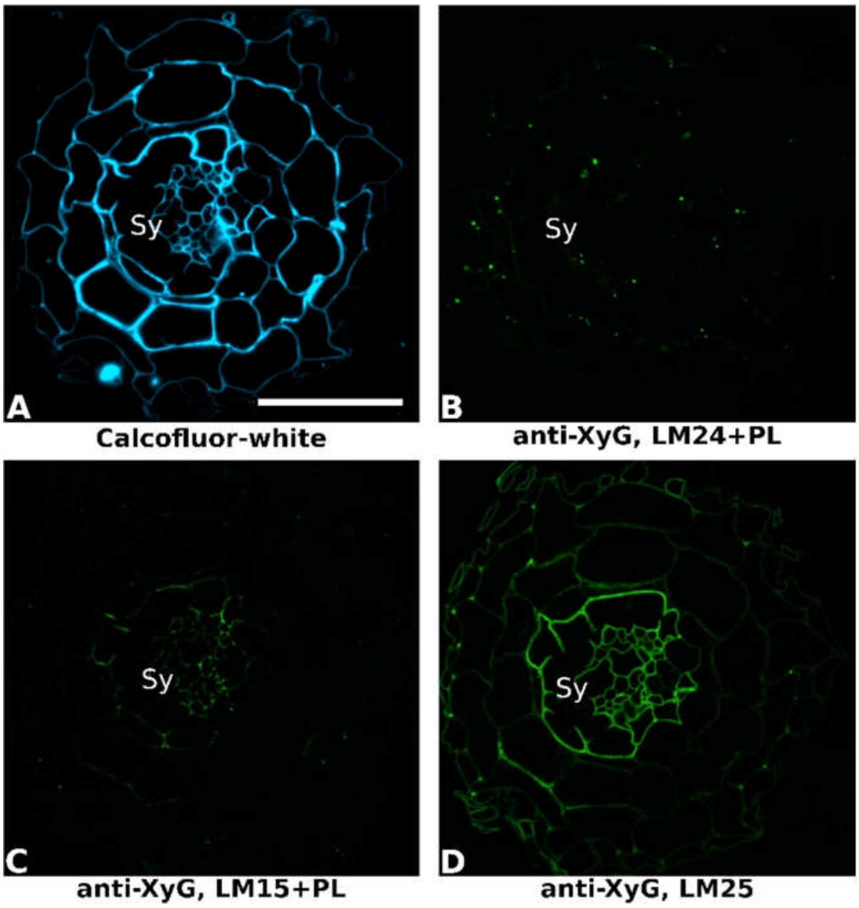

Figure S3. Immunofluorescence imaging of a syncytium induced by potato cyst nematode *Globodera pallida* within a potato root (cv Desiree, 14 dpi). (A) Section stained with Calcofluor White for visualization of all cell walls. Indirect immunofluorescence (green) resulting from the binding of specific mAbs is shown for corresponding serial sections: **(B)** LM24 to xyloglucan (XyG); **(C)** LM15 to xyloglucan (XyG); **(D)** LM25 to to xyloglucan (XyG). Sy indicates location of the syncytium. PL = section pretreated with pectate lyase. Scale bar = 50 µm.

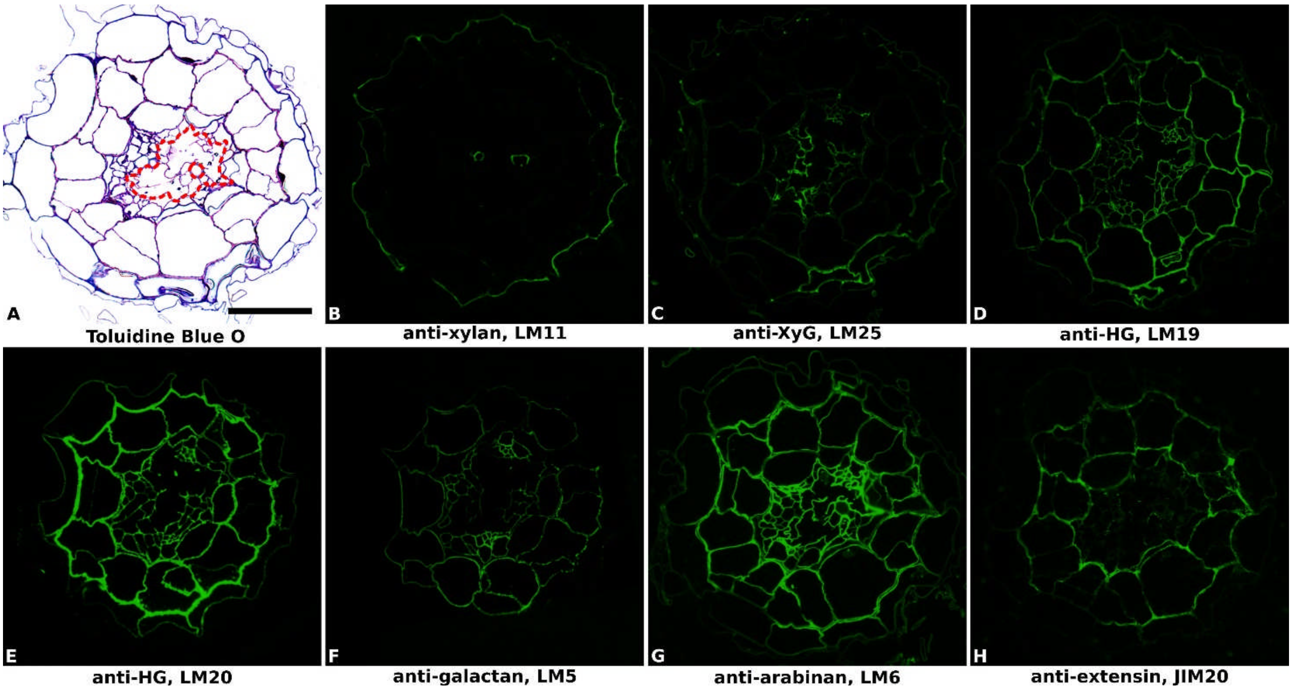

Figure S4. Immunofluorescence imaging of a sectioned potato root infected with *G. pallida* (cv Desiree, 7 dpi). (A) The extent of the syncytium is indicated with a red line in the Toluidine Blue O stained bright field image. Indirect immunofluorescence (green) resulting from the binding of specific mAbs is shown for corresponding serial sections: **(B)** LM11 to heteroxylan; **(C)** LM25 to xyloglucan (XyG); **(D)** LM19 to non/low methyl-esterified homogalacturonan (HG); **(E)** LM20 to methyl-esterified HG; **(F)** LM5 to pectic galactan; **(G)** LM6 to arabinan; **(H)** JIM20 to extensin. Within the vascular cylinder LM11 binds only to the xylem vessels **(B)** so serves to identify the location of these cells in all sections. Scale bar = 5

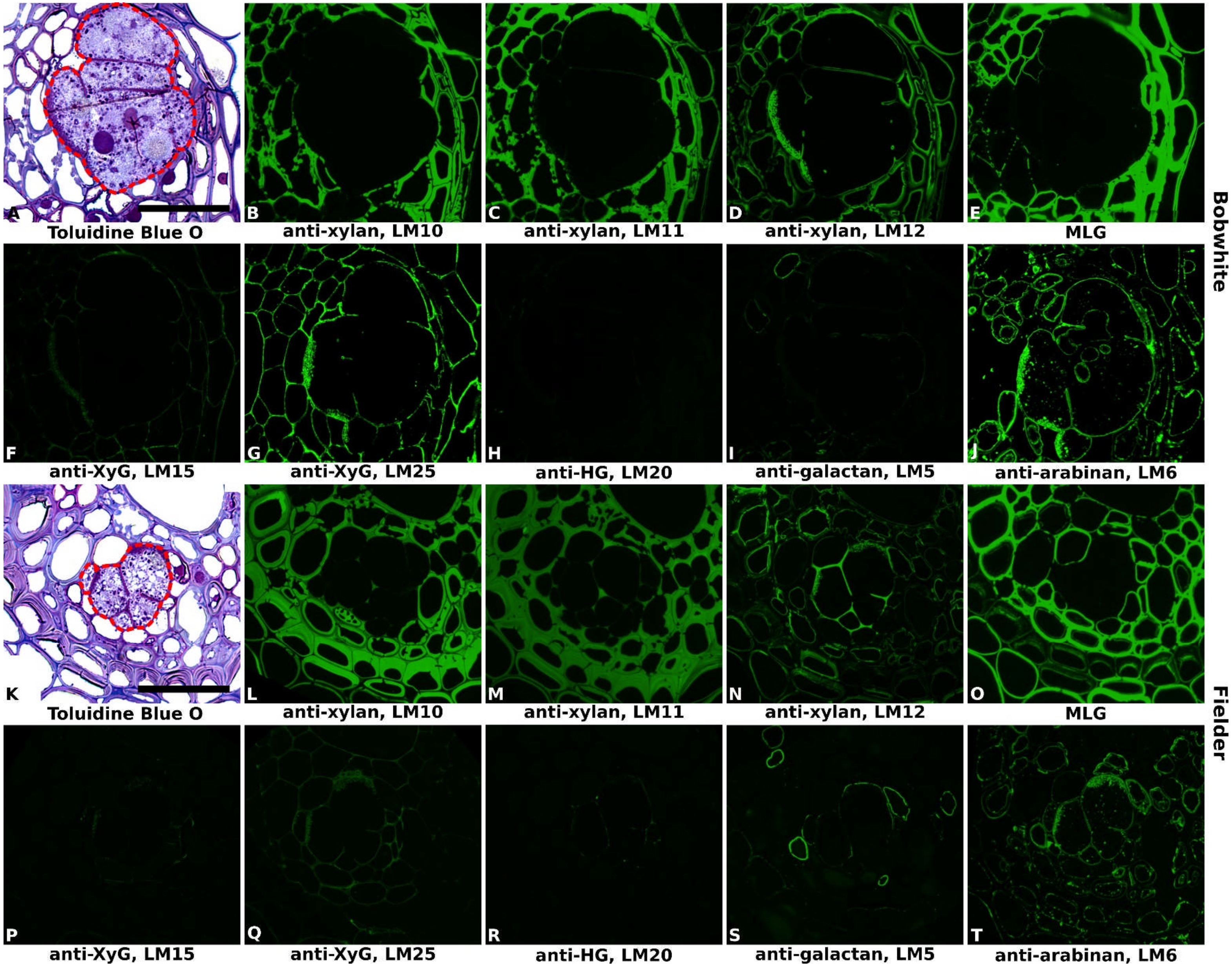

Figure S5. Immuno-fluorescence imaging of syncytia induced by *H. avenae* within wheat roots (cv Bobwhite and Fielder, 21 dpi). (A for cv. Bobwhite & K for cv. Fielder) The extent of the syncytia are indicated with a red line in the Toluidine Blue O stained bright field images. Indirect immunofluorescence (green) resulting from the binding of specific mAbs is shown: **(B & L)** LM10 to heteroxylan; **(C & M)** LM11 to heteroxylan; **(D & N)** LM12 to feruloylated heteroxylan; **(E & O)** MLG to mixed linkage glucan; **(F & P)** LM15 to xyloglucan (XyG); **(G & Q)** LM25 to XyG; **(H & R)** LM20 to methyl-esterified homogalacturonan (HG); **(I & S)** LM5 to pectic galactan; **(J & T)** LM6 to arabinan. Scale bar = 50 µm.

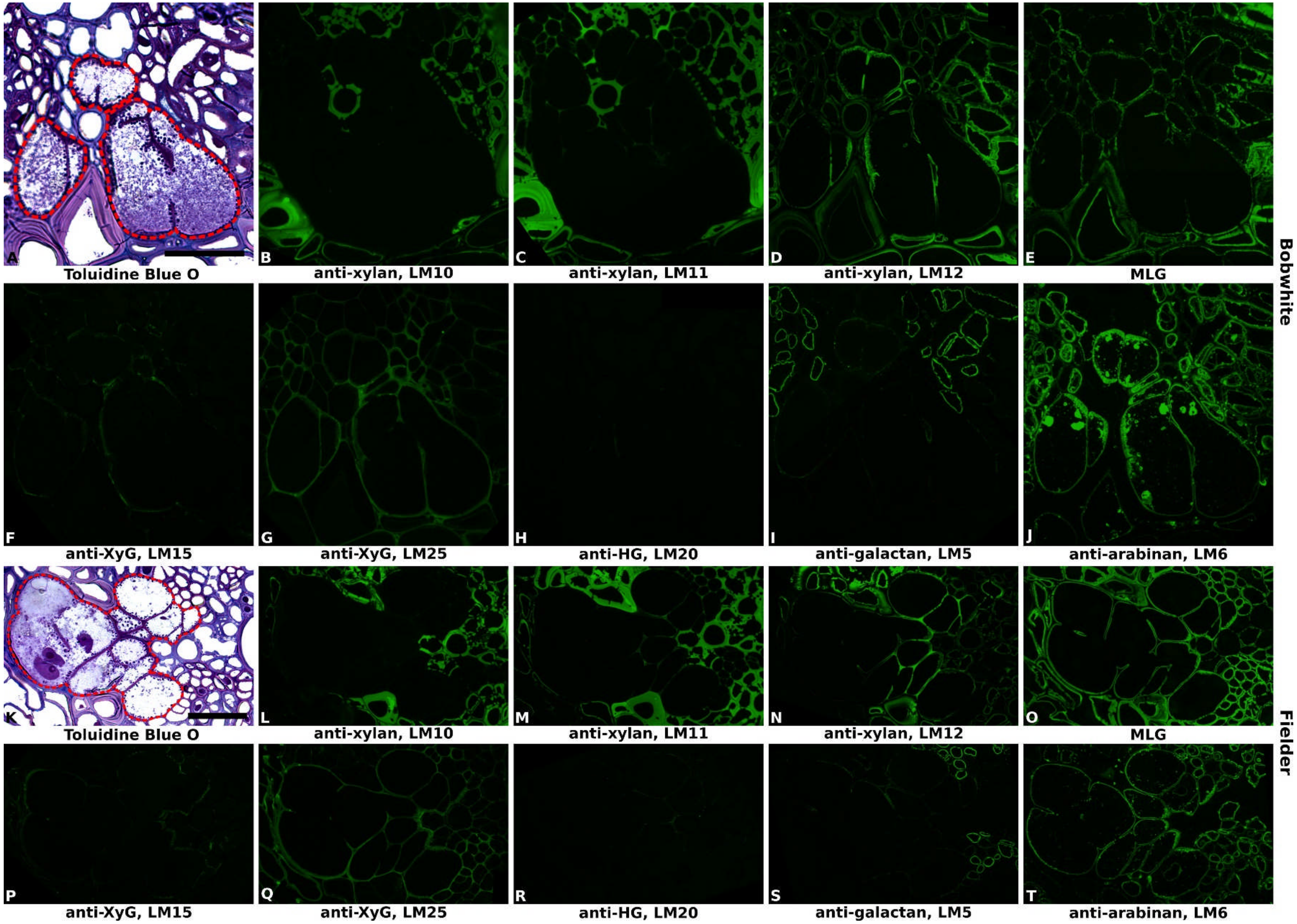

Figure S6. Immunofluorescence imaging of syncytia induced by *H. filipjevi* within wheat roots (cv Bobwhite and Fielder, 28 dpi). (A for cv. Bobwhite & K for cv. Fielder) The extent of the syncytia are indicated with a red line in the Toluidine Blue O stained bright field images. Indirect immunofluorescence (green) resulting from the binding of specific mAbs is shown: **(B & L)** LM10 to heteroxylan; **(C & M)** LM11 to heteroxylan; **(D & N)** LM12 to feruloylated heteroxylan; **(E & O)** MLG to mixed linkage glucan; **(F & P)** LM15 to xyloglucan (XyG); **(G & Q)** LM25 to XyG; **(H & R)** LM20 to methyl-esterified homogalacturonan (HG); **(I & S)** LM5 to pectic galactan; **(J & T)** LM6 to arabinan. Scale bar = 50 µm.

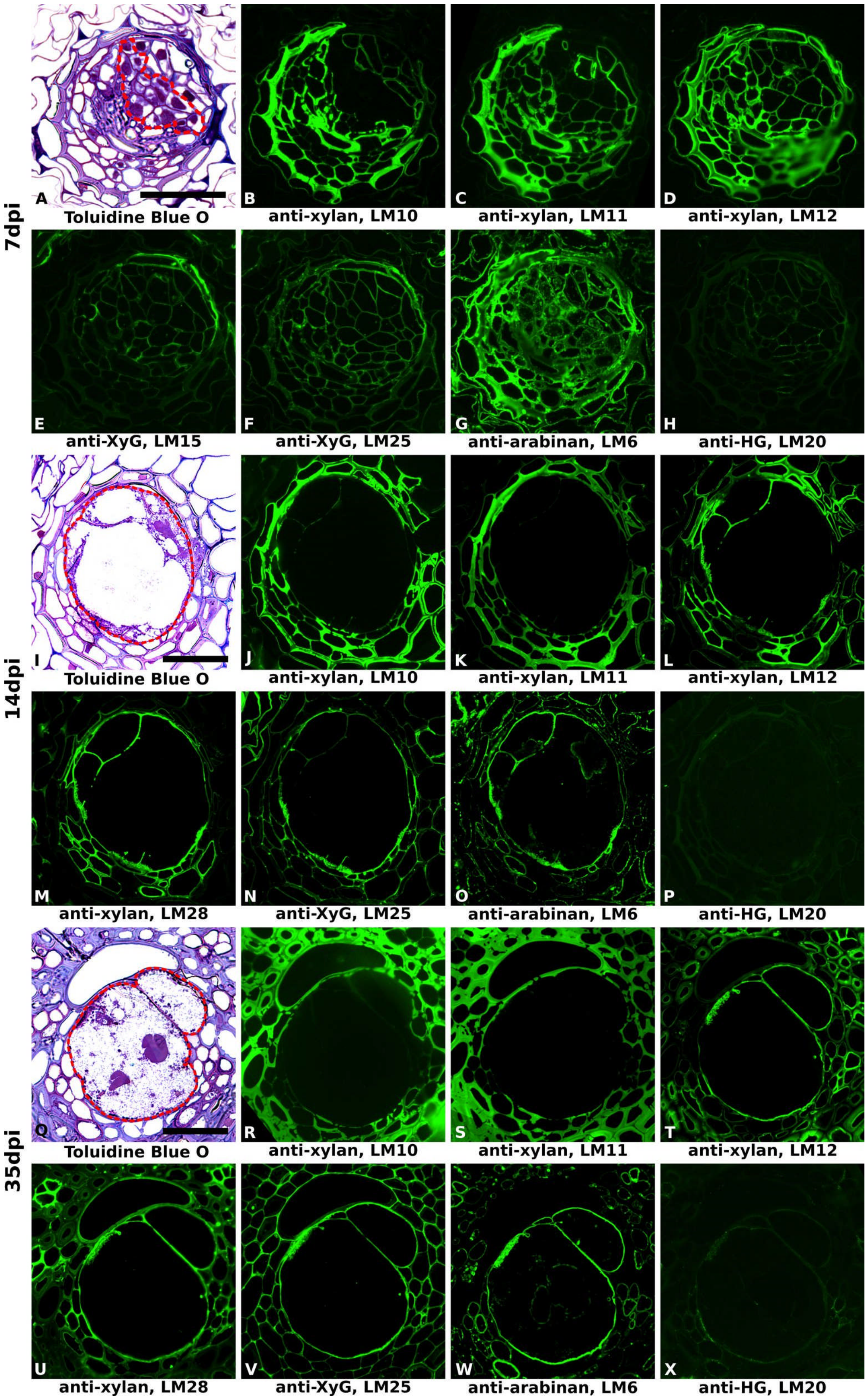

Figure S7. Immuno-fluorescence imaging of syncytia induced by *H. avenae* within roots of wheat cv. Bobwhite at different time points post infection. (A, I, Q) Syncytial regions are circled with a red line in the Toluidine Blue O stained (bright field) images. Indirect immunofluorescence (green) resulting from the binding of specific mAbs is shown: **(B, J & R)** LM10 to heteroxylan; **(C, K & S)** LM11 to heteroxylan; **(D, L & T)** LM12 to feruloylated heteroxylan; **(E)** LM15 to xyloglucan (XyG); **(F, N & V)** LM25 to xyloglucan (XyG); **(G, O & W)** LM6 to arabinan; **(H, P & X)** LM20 to methyl-esterified homogalacturonan (HG); **(M & U)** LM28 to glucuronosyl-containing heteroxylan. Scale bars = 50µm.

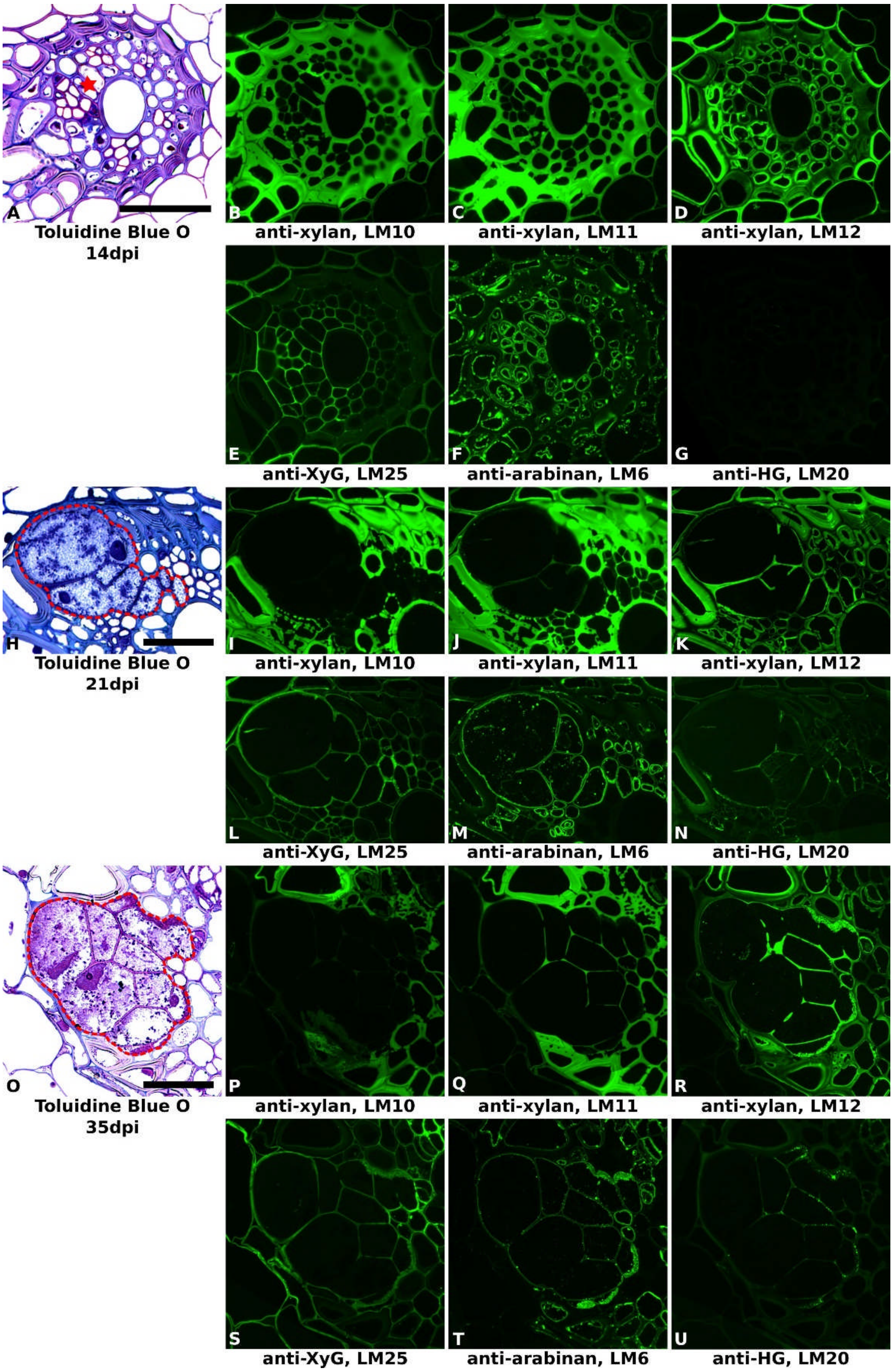

Figure S8. Immunofluorescence imaging of syncytia induced by *H. filipjevi* within roots of wheat cv. Bobwhite at different time points post infection. **(A)** The likely developing syncytium is indicated by a red star in the Toluidine Blue O bright field image at 7 dpi; **(H, O)** Syncytial regions are circled with a red line in the Toluidine Blue O stained (bright field) images at 21 & 35 dpi. Indirect immunofluorescence (green) resulting from the binding of specific mAbs is shown: **(B, I & P)** LM10 to heteroxylan; **(C, J & Q)** LM11 to heteroxylan; **(D, K & R)** LM12 to feruloylated heteroxylan; **(E, L & S)** LM25 to xyloglucan (XyG); **(F, M & T)** LM6 to arabinan; **(G, N & U)** LM20 to methyl-esterified homogalacturonan (HG). Scale bars = 50µm.
